# Comparison of diagnostic methods for the detection and quantification of the four sympatric *Plasmodium *species in field samples from Papua New Guinea

**DOI:** 10.1186/1475-2875-9-361

**Published:** 2010-12-14

**Authors:** Anna Rosanas-Urgell, Dania Mueller, Inoni Betuela, Céline Barnadas, Jonah Iga, Peter A Zimmerman, Hernando A del Portillo, Peter Siba, Ivo Mueller, Ingrid Felger

**Affiliations:** 1 Papua New Guinea Insitute of Medical Research, Madang 511, Papua New Guinea; 2 Barcelona Centre for International Health Research (CRESIB), Hospital Clinic/IDIBAPS, Universitat de Barcelona; Roselló 132, 4a planta, 08036, Barcelona, Spain; 3 Swiss Tropical and Public Health Institute, Socinstrasse 57, CH-4002 Basel, Switzerland; 4 University of Basel, Petersplatz 1, CH-4003 Basel, Switzerland; 5 Center for Global Health and Diseases, Case Western Reserve University School of Medicine, Cleveland, Ohio; 6 Institució Catalana de Recerca i Estudis Avançats (ICREA), Barcelona, Spain

## Abstract

**Background:**

Accurate diagnosis of *Plasmodium *infections is essential for malaria morbidity and mortality reduction in tropical areas. Despite great advantages of light microscopy (LM) for malaria diagnosis, its limited sensitivity is a critical shortfall for epidemiological studies. Robust molecular diagnostics tools are thus needed.

**Methods:**

The present study describes the development of a duplex quantitative real time PCR (qPCR) assay, which specifically detects and quantifies the four human *Plasmodium *species. Performance of this method was compared to PCR-ligase detection reaction-fluorescent microsphere assay (PCR_LDR_FMA), nested PCR (nPCR) and LM, using field samples collected from 452 children one to five years of age from the Sepik area in Papua New Guinea. Agreement between diagnostic methods was calcualted using kappa statistics.

**Results:**

The agreement of qPCR with other molecular diagnostic methods was substantial for the detection of *P. falciparum*, but was moderate for the detection of *P. vivax*, *P. malariae *and *P. ovale*. *P. falciparum *and *P. vivax *prevalence by qPCR was 40.9% and 65.7% respectively. This compares to 43.8% and 73.2% by nPCR and 47.1% and 67.5% by PCR_LDR_FMA. *P. malariae *and *P. ovale *prevalence was 4.7% and 7.3% by qPCR, 3.3% and 3.8% by nPCR, and 7.7% and 4.4% by PCR_LDR_FMA. Prevalence by LM was lower for all four species, being 25.4% for *P. falciparum*, 54.9% for *P. vivax*, 2.4% for *P. malariae *and 0.0% for *P. ovale*. The quantification by qPCR closely correlated with microscopic quantification for *P. falciparum *and *P. vivax *samples (R2 = 0.825 and R2 = 0.505, respectively). The low prevalence of *P. malariae *and *P. ovale *did not permit a solid comparative analysis of quantification for these species.

**Conclusions:**

The qPCR assay developed proved optimal for detection of all four *Plasmodium *species. Densities by LM were well reflected in quantification results by qPCR, whereby congruence was better for *P. falciparum *than for *P. vivax*. This likely is a consequence of the generally lower *P. vivax *densities. Easy performance of the qPCR assay, a less laborious workflow and reduced risk of contamination, together with reduced costs per sample through reduced reaction volume, opens the possibility to implement qPCR in endemic settings as a suitable diagnostic tool for large epidemiological studies.

## Background

Rapid and accurate diagnosis of *Plasmodium *infections is crucial for morbidity and mortality reduction in tropical areas, specially in regions where mixed infections are prevalent such as Papua New Guinea (PNG), where all four parasites infecting humans coexist and mixed species infections are common [1,2]. For improving accuracy in large epidemiological studies, molecular diagnostic tools permitting high through put analysis for the identification and quantification of malaria parasites would be of great benefit.

Traditionally, light microscopy (LM) examination of blood smears has been considered the gold standard for the diagnosis of malaria [3]. LM has clear advantages over molecular typing, since it incurs only low costs, does neither need complex sample preparation nor advanced technology and permits species identification and quantification [4]. However, the role of LM as a gold standard has been questioned due to false negative results at low parasitemia and frequent errors in species identification in mixed infections [5,6].

The availability of commercial rapid diagnostic tests (RDT) has greatly facilitated the in situ diagnosis of malaria infections in the field. The advantages of RDT are that they do not need special preparation of the sample and diagnostic results become immediately available [7]. However, their use is limited due to lack of sensitivity for *Plasmodium vivax, Plasmodium malariae *and *Plasmodium ovale *[8].

Nested PCR (nPCR), first described by Snounou and co-workers [9], is a widely used method and is considered as a molecular gold standard due to its good performance in detection of mixed species infection. This assay amplifies the multicopy 18 S rRNA genes of the four *Plasmodium *species infecting humans. Even though this genotyping technique is now performed in many field laboratories in endemic countries, its use for routine clinical diagnosis is limited, because the analysis is time consuming due to the need of multiple reactions per sample, and the risk of contamination through the requirement for nPCR [9]. Moreover, the technique is not quantitative.

In recent years, various real time quantitative PCR (qPCR) assays have been developed for the detection of *Plasmodium *species, with most assays targeting the 18 S rRNA genes. Two of these methods detect the genus *Plasmodium *using generic primers, and thus do not distinguish between species [10,11]. Sybergreen reagent has been used by other groups, to identify the four *Plasmodium *species infecting humans in a single reaction by melting curve analysis [8,12]. The use of TaqMan probes contributed an additional level of specificity to qPCR assays [13]. However, using a single pair of primers for *Plasmodium *genus detection in duplex assays, introduces competition for amplification among species, which likely leads to difficulties in detecting mixed infections [14]. In order to address this issue, various groups had used different strategies [15-18]. The multiplex PCR-ligase detection reaction-fluorescent microsphere assay (PCR_LDR_FMA) has also been used in molecular epidemiological studies for simultaneously detecting all four *Plasmodium *species [19].

Despite the variety of molecular tools available for the diagnosis of malaria and their wide use for the diagnosis of imported malaria in travel clinics, implementation of these techniques in endemic areas has remained limited until now. Even though the low sensitivity and limited detection of asymptomatic and mixed infections by LM constitutes a critical shortfall for some epidemiological studies, LM remains to date the most frequently used method for the diagnosis of malaria in endemic areas. The transfer of molecular techniques for diagnosis of malaria to laboratories in endemic settings is essential for overcoming the limitations by LM. Moreover, a molecular technique with quantification capacity contributes to correctly estimate the burden of *Plasmodium *species often found in concomitant infections and will be a valuable tool to explore competition in mixed infections.

A qPCR assay initially developed for malaria diagnosis in returning travellers at a reference laboratory was implemented and validated at the PNG Institute of Medical Research (IMR). This assay was chosen because it detects with high specificity all four *Plasmodium *species which jointly occur in our study area in PNG. The performance of this qPCR assay in conditions of a field laboratory and on field samples was compared to light microscopy, nPCR and PCR_LDR_FMA results.

## Methods

### Study site and sample collection

Samples were collected in 10 villages from Ilahita area of East Sepik Province, PNG [2], in the framework of a longitudinal cohort study conducted during 2008. As part of the baseline, venous blood samples were obtained from 452 children one to five years of age, after written informed consent was obtained from parents or legal guardians of each child. This genotyping study was approved by the PNG IMR Institutional Review Board (IMR IRB 0720) and PNG Medical Research Advisory Committee (MRAC 07.34).

### Blood smear examination

Thick/thin blood smears were prepared as described previously [20,21]. Blood smears were stained with a 5% Giemsa solution and examined independently by two microscopists, with a third microscopist reading for those slides with discrepant results. A minimum of 200 microscopic fields were examined at a magnification of 1000× using oil immersion optics before a slide was declared negative for malaria parasites by LM. *Plasmodium *species were quantified by counting infected parasites over 200 leukocytes. Conversion of parasite counts into parasites/μL was performed assuming a mean leukocyte count of 8000/μL whole blood. Routinely slides were read twice. Discordant results were evaluated by a third slide reading. Final species diagnosis was based on the majority agreement.

### DNA template extraction and amplification

DNA was extracted from 200 μL whole blood (venous blood collected in EDTA anti-coagulant) using QIAamp 96 DNA Blood Mini Kit (QIAGEN, Valencia, CA), and eluted in a final volume of 200 μL dH2O according to the supplier's instructions. nPCR was carried out as described [22].

PCR_LDR_FMA was carried out as described elsewhere [19]. Mixes of serially diluted plasmids containing inserts of *P. falciparum*, *P. vivax*, *P. malariae *or *P. ovale *18 S rDNA were used as positive controls in addition to *P. falciparum *or *P*. *vivax *positive samples obtained from field isolates. The threshold for positivity for each species was determined using the mean value obtained from negative controls for each species, plus three times the standard deviation.

The primers and probes of the qPCR assay are listed in Table 1. In the design of a duplex qPCR, the probes combined in one reaction carried different fluorescent labels at their 5' ends. All four probes carried a black hole quencher (BHQ) at their 3'ends. The analytical specificity of primers and probes were evaluated for each *Plasmodium *species *in silico *by Blast searches and experimentally by using gDNA of the three alternatives *Plasmodium *species or of related blood borne parasites. To minimize costs of consumables, duplex reactions were performed in a final volume of 12.5 μL. Amplification and detection of the amplified product was performed in an iQcycling BioRad system, using iQSupermix from BioRad. The *P. falciparum/P. vivax *(*Pf/Pv*) duplex reaction contained 2.5 μL DNA (corresponding to 2.5 μL whole blood), 6.25 μL SuperMix iQ (BioRad), 0.35 μL *Pf *primer mix (50 μM), 0.35 μL of *Pv *primer mix 50 μM, 0.375 μL of *Pf *probe (10 μM), 0.375 μL of *Pv *probe (10 μM) and 2.3 μL double distilled water. The *P. malariae/P. ovale *(*Pm/Po*) duplex reaction contained equivalent amounts and concentrations of the respective primers and probes. The thermal profile used was 2 minutes at 50°C, followed by 10 minutes at 95°C and 45 cycles of 15 seconds at 95°C and 1 minute at 58°C.

**Table 1:** Primers and probes for qPCR

Species	Primer/ probe	Sequence 5'-3'	Fluorescent label
*P. falciparum*	Fal_F	TATTGCTTTTGAGAGGTTTTGTTACTTTG	
*P. falciparum*	Fal_R	ACCTCTGACATCTGAATACGAATGC	
*P. falciparum*	Fal_P	ACGGGTAGTCATGATTGAGTT	FAM-BHQ
*P. vivax*	Viv_F	GCTTTGTAATTGGAATGATGGGAAT	
*P. vivax*	Viv_R	ATGCGCACAAAGTCGATACGAAG	
*P. vivax*	Viv_P	AGCAACGCTTCTAGCTTA	HEX-BHQ
*P.malariae*	Mal_F	TGCCGACTAGGTGTTGGATGAT	
*P.malariae*	Mal_R	CTAGTGAGTTTCCCCGTGTTGAGT	
*P.malariae*	Mal_P	TGTTTCTTTTAGATAGCTTCCTTCAG	FAM-BHQ
*P. ovale*	Ova_F	CCAGCTCCAATAGCGTATATTAAA	
*P. ovale*	Ova_R	ACACATTTTGSATAAGGAATGCAAAG*	
P. ovale	Ova_P	TATAAGATGCTTAGRCAATACAACGTATCTG*	HEX-BHQ

*Oligo includes a wobble (S: G or C; R: A or G)

### qPCR validation

Evaluation of PCR efficiency and reproducibility was performed on standard curves using four positive control plasmids with the respective amplicons inserted. Geometric mean and standard deviation were calculated from triplicates in three independent assays. Standard curve for each *Plasmodium *species was made from a 10-fold serial dilution of the control plasmids ranging from 10^6 ^copies/μL to 10 copies/μL.

Amplification efficiencies for the different primer pairs and probes were calculated with the formula:

Efficiency = 10^(-1/Slope) ^-1. Inter-assay coefficients of variation (CVs) were calculated for each plasmid dilution separately as (SD/mean)*100 using the Ct values from different runs.

Reproducibility of qPCR was further analysed by repeating 10% of all 452 field-samples in the same laboratory, but at different time points. External quality assurance was performed in addition.

The amount of target in an unknown sample was quantified by converting the threshold cycle (Ct) into template copy number by using the four standard curves. Samples yielding Ct values equal or higher than 40 were considered *Plasmodium *species negative [23].

### Statistical analysis

Results from qPCR was compared with those from LM, nPCR and PCR-LDR-FMA. Agreement between diagnostic methods was determined by calculating Kappa Statistics with 95% confidence intervals. Values were interpreted with the Landis and Koch classification [24] as follows: k = 0.41-0.60, moderate agreement, k = 0.61-0.80, good agreement; k = 0.81-1.00, almost perfect agreement beyond chance. Prevalence for each species was calculated by dividing the number of positive samples through by the number of all samples tested and McNemar test was calculated in order to test for significance. *P *values <0.05 were considered statistically significant. Correlation between LM and qPCR quantification was calculated by pairwise correlation coefficient. All statistical calculations were performed with STATAv.10 statistical software.

## Results

### Optimization of qPCR assay

The large scale of field work required optimization of molecular testing with the aim to reduce costs. Two strategies were applied: multiplexing the qPCR and reducing the reaction volume. The performance of the two duplex qPCRs were evaluated by analysing dilution series of the two respective control plasmids in various ratios (Additional file 1). These experiments demonstrated very little inhibition when 200 templates and above were present in the reaction. Only the detection of minute template concentrations, as low as 20 templates per reaction, was slightly inhibited by excess amounts of the alternative template, i.e. in a 100 times excess.

Table 2 shows the duplex qPCR efficiencies for all four templates after reducing the reaction volume to 12.5 μL. The dilution series was performed with control plasmids containing inserts corresponding to the *Plasmodium *species-specific PCR product. Similar slopes were observed for all species, demonstrating similar amplification efficiency throughout the tested range and therefore comparability between quantification results. Correlation coefficients around 0.980 demonstrated that our assays were linear over the entire quantification range. The inter-assay coefficients of variation (CV) between 0.3% and 5%, obtained in all six dilutions, indicated reproducible results (Additional file 2).

**Table 2:** qPCR Efficiencies for each Plasmodium species

	Slope	Efficiency %	Interception	R squared
*P. falciparum*	-3.50	93.17%	39.33	0.987
*P. vivax*	-3.59	89.7%	38.56	0.975
*P. malariae*	-3.57	90.46%	40.26	0.989
*P. ovale*	-3.51	92.54%	38.83	0.988

Reproducibility of our assay was investigated by re-analysing 10% of the 452 samples in the same laboratory. In 8/45 compared samples discordant results were recorded. Four of these discrepancies occurred in cases of low density in the range of 10-100 copies/μL. Ct values were between 37 and 39 in the positive ones of the duplicate tests. Two other discrepancies were observed in mixed species infections, whereby the minority species was detected in only one of the duplicate experiments in the range of 10 to 100 copies/μL.

### Comparison of diagnosis of *Plasmodium *infection by qPCR, nPCR, PCR-LDR and LM

Prevalence values for *P. falciparum *showed significant differences when all detection techniques were considered (40.9% by qPCR, 43.8% by nPCR and 47.1% by LDR, p-values < 0.05). However, greater agreement was observed for qPCR compared to LM (k = 0.621) or to PCR-LDR (k = 0.750). Almost perfect agreement was obtained for prevalence by qPCR and by nPCR (k = 0.869) (Table 3). Prevalence for *P. vivax *by qPCR was not significantly different from that by LDR (65.7% *versus *67.5%, p-value > 0.5), but a statistically significant difference was seen when comparing to nPCR (prevalence 73.2%, p-value < 0.05) or LM (prevalence 54.9%, p-value < 0.05). Overall, *P. vivax *prevalence by qPCR was in moderate agreement with the other assays (0.596 with nPCR, 0.553 with PCR-LDR and 0.530 with LM). Prevalence values for *P. malariae *and *P. ovale *were highly discrepant between the different techniques. Prevalence of *P. malariae *by qPCR (4.7%) differed significantly from that of PCR-LDR (prevalence 7.7%, p-value < 0.05), and from that of nPCR (prevalence 3.3%, p-value < 0.05). Moreover, prevalence of *P. ovale *was higher by qPCR (7.3%) compared to that of LDR (4.4%) and nPCR (3.8%). The difference between all these values was statistically significant. Prevalence by LM was significantly lower for all four species (25.4% for *P. falciparum*, 54.9% for *P. vivax*, 2.4% for *P. malariae *and 0% for *P. ovale*, p-values < 0.05). *P. malariae *and *P. ovale *moderate kappa scores were retrieved when comparing qPCR with the other assays (Table 4).

**Table 3:** P. falciparum versus P. vivax agreement among different diagnostic techniques

	qPCR	nPCR	PCR-LDR	LM
**qPCR**		0.869 perfect	0.750 substantial	0.621 substantial
**nPCR**	*0.596* *moderate*		0.737 substantial	0.562 moderate
**PCR-LDR**	*0.553 moderatee*	*0.609 substantial*		0.517 moderate
**LM**	*0.530* *moderate*	*0.513* *moderate*	*0.611 substantial*	

*P. falciparum *(normal case). *P. vivax *(*italics*).

**Table 4:** P. malariae versus P. ovale agreement among different diagnostic techniques

	qPCR	nPCR	PCR-LDR	LM
**qPCR**		0.595 moderate	0.545 moderate	0.484 moderate
**nPCR**	*0.621* *substantial*		0.455 moderate	0.446 moderate
**PCR-LDR**	*0.541* *moderate*	*0.577* *moderate*		0.458 moderate

*P. malariae *(normal case). *P. ovale *(*italics*).

### Quantification of *Plasmodium *parasites

**Figure 1:**
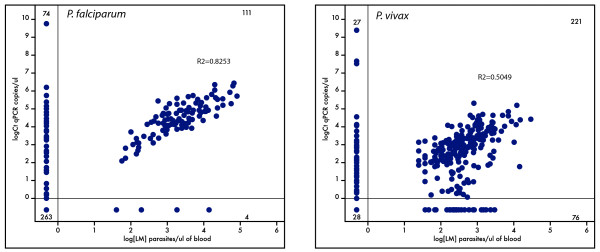
**Comparison of quantification assessed by light microscopy versus qPCR**.

For *P. falciparum*, quantification by qPCR correlated well with microscopy counts when both were positive (R^2 ^= 0.8253). A substantial number of samples were negative for *P. falciparum *by LM, but positive by qPCR; in the scatter blot in Figure 1A these negative samples locate to the ordinate. Very few samples were LM positive, but qPCR negative for *P. falciparum*. As the densities of these samples were not extremely low and all of them were co-infected with other *Plasmodium *species, microscopic misclassification of the *Plasmodium *species is likely. The correlation between microscopy counts and qPCR-based densities was lower for *P. vivax *(R^2 ^= 0.5049). This lower agreement might be due to overall lower densities, possibly around the detection limit, in *P. vivax *infections. Such stochastic variation in parasite presence likely was responsible for negative slide results as well as for a negative qPCR, both indicated by numerous data points on the coordinates in Figure 1B. Generally in all PCR-based techniques low densities lead to alternating positive or negative results in repeated experiments due to the coincidental lack of any template in some reactions. Furthermore, mixed species infections, where *P. vivax *is found at a very low density, contribute to a lower correlation between microscopy count and qPCR-based densities likely due to misclassification by LM. To illustrate this, *P. vivax *correlation coefficient significantly increased up to R^2 ^= 0.7220 when only single infections by qPCR are analysed, but was as low as R^2 ^= 0.4372 when only mixed infections are considered. Few of the samples positive for *P. malariae *by qPCR and none of the *P. ovale *positive samples were identified by light microscopy.

## Discussion

In preparation of major molecular epidemiological field studies in PNG essential parasite detection techniques were compared under conditions of a laboratory close to the field site and located in a malaria endemic country. The diagnostic requirements were: (i) good performance in the detection of mixed species infections, as all four species concurrently occur in PNG, (ii) recognition of *P. malariae *and *P. ovale *variants present in the study area, (iii) high through put capacity and robustness of assay, (iv) quantitative results and (v) reasonable costs. The qPCR assay described here was implemented and validated at the PNG-IMR site in Madang, demonstrating the feasibility of applying state of the art techniques in this context. In the meantime the qPCR assay is routinely implemented for molecular diagnosis in large scale epidemiologic studies at IMR.

As part of test validation in the field, the performance of this qPCR assay for Plasmodium species discrimination was compared to two other PCR-based assays (nPCR and LDR) and to LM. Traditionally, test outcomes for different assays are compared to an established 'gold-standard' in order to calculate sensitivity and specificity estimates and to evaluate the performance of newly developed tests. The classical 'gold standard' for malaria diagnosis has been LM [3], however, with the appearance of new molecular diagnostic tools, LM has become less suitable for this purpose due to its lower sensitivity than molecular methods [6]. Even though the nPCR developed by Snounou et al [9] has been extensively used as 'gold-standard' for molecular diagnosis [25,26], the concept of using a 'gold-standard' for the evaluation of new assays is being questioned by various authors, which alternatively propose the use of 'non-gold standard' approaches [27,28].

The agreement between qPCR and the other techniques was substantial for *P. falciparum*, but only moderate for *P. vivax*, *P. malariae *and *P. ovale*. In particular, the agreement between qPCR and nPCR for *P. falciparum *detection was almost perfect. The lower agreement between PCR-LDR and nPCR, together with the higher prevalence shown by PCR-LDR (47.1% compared to 40.9% by qPCR and 43.8% by nPCR), may indicate false positive results by LDR. This is supported by our pairwise analysis and the agreement of two independent PCR based assays, namely nPCR and qPCR. However, in absence of a suitable diagnostic 'gold standard', it remains unclear if those 33 samples positive for LDR but negative by the two alternative molecular methods, represent a greater sensitivity of LDR or simply false positives. This issue cannot be easily resolved in a study involving 'unknown' samples from the field, potentially infected by four different *Plasmodium *species.

*P. vivax *prevalence was higher by nPCR than by both, qPCR and PCR-LDR (73.2% by nPCR, 65.7% by qPCR and 67.5% by PCR-LDR). This again could reflect false postitives by nPCR or lower sensitivity by both other molecular methods. Our observations in qPCR validation using plasmid template suggested that qPCR of *P. vivax *is lightly compromised by performing a duplex *Pf/Pv *reaction. nPCR involves a very high number of cycles (55 cycles by nPCR versus 45 cycles by qPCR and 35 cycles by LDR), and therefore is expected to show maximal sensitivity. Despite measurements taken over 45 cycles in qPCR, we followed the consensus rule for considering a sample positive, i.e. a Ct value < 40 [23]. In our samples this led to the loss of 9 samples with Ct values for *P. vivax *between 40 and 43.6 cycles, which otherwise would have increased the sensitivity of the assay. Further analysis was performed on samples with discrepant results for *P. vivax *(*P. vivax *negative samples by qPCR and positive by nPCR). Most of these samples were mixed infections by nPCR and harboured *P. falciparum *with more than 10,000 copies/μl. Thus competition for amplification at the beginning of the PCR due to *P. falciparum *high densities may be precluding *P. vivax *detection [14]. 14/16 of the remaining samples were also negative by LM. Most likely these very low-grade *P. vivax *infections were missed. The scarcity of the template in case of a very low parasite density is expected to lead to imperfect detection. Prevalence for *P. malariae *and *P. ovale *were low with significant differences between assays, even though the agreement between pairwise compaired methods was moderate. Higher prevalence for *P. malariae *detection by LDR is likely to occur as a result of false positive results, probably occurring due to high background noise of the *P. malariae *probe used in the assay. Low detection of *P. ovale *by nPCR (3.8%) is due to the use of a primer pair with sub-optimal amplification of *P. ovale *sequences present in the study area. Finally, LM measured the lowest prevalence for all four *Plasmodium *species.

The major advantage of qPCR over the other compared molecular techniques was the quantification of parasite densities. Parasite densities shown as copies of 18 S rRNA template/μL were quantified by converting the threshold cycle (Ct) into template copy number by using the standard curves. When correlating quantification by qPCR with LM counts in samples where both techniques showed positive results, a high correlation for *P. falciparum *(R^2 ^= 0.8253) and a lower correlation for *P. vivax *(R^2 ^= 0.5049) was found. But for *P. vivax *this correlation of parasite densities by qPCR and LM increased when only single infections were taken into account. Therefore, our results suggest a variation in the detection limit in both methods, due to overlooking *P. vivax *in case of an overwhelming *P. falciparum *infection. Difficulties in identifying *P. vivax *by LM arise when this parasite is found at low densities and in mixed infections. The high *P. falciparum *densities found in the samples identified as mixed infection by qPCR (> 10000 target copies/μl) further supports this explanation. The correlation for *P. malariae *and *P. ovale *could not be analysed due to poor detection of both species by LM.

The qPCR assay was found optimal for both tasks, detection of all four *Plasmodium *species and quantification. The latter could only be analyzed for *P. falciparum *and *P. vivax*. Overall qPCR shows substantial agreement with other molecular techniques for the detecting prevalence of *P. falciparum *and *P. vivax*, while moderate agreement was observed for *P. malariae *and *P. ovale*. It is clear, that sensitivity of our qPCR assay can be increased by simply performing independent reactions of each *Plasmodium *species. However, this would substantially increase costs. Limiting factors, such as duplex assays, need to be balanced against costs or work load. The specific research objectives of a particular study should guide the choice of experimental procedures.

Overall, the superior performance of PCR based methodologies over LM has been clearly demonstrated by these results and others. In a recent study conducted in Benin, a high number of children (between 27% and 44%) aged 5 or above, who initially had negative RDT tests (most also with negative blood slides), were later found to be infected with *P. falciparum *using PCR [29]. These undetected submicroscopic infections have an enormous impact for malaria transmission in endemic areas. In a time where malaria erradication has become the primary goal of malaria agendas, the accurate estimation of the burden of malaria infection is imperative to control transmission.

## Conclusions

In conclusion, this qPCR assay was sensitive and specific for the detection of all four *Plasmodium *species and results agreed well with other molecular techniques tested. Added advantage of the qPCR assay is quantification of parasite densities and a less laborious workflow. Moreover, the assay performed well in field samples and due to its high through put capacity, it is suitable for large scale epidemiological studies. Finally, a quantitative assay is of a greatest value for monitoring of malaria control programmes.

## Competing interests

The authors declare that they have no competing interests.

## Authors' contributions

DM and IF conceived and designed qPCR assay and AR coordinated the study. DM validated qPCR in the reference lab and AR in the endemic seating. IB and AR participated in the sample collection. JI did the DNA extractions from the blood samples. CB performed the PCR-LDR-FMA. AR performed qPCR and nPCR methods, statistical analysis and interpretation of the data. AR, IF and IM draft the manuscript. PZ and HP critically reviewed the manuscript. All authors have read and approved the final manuscript.

## Supplementary Material

Additional file 1 **Table S1. Effect of mixed-species infection on sensitivity of duplex qPCR**. Click here for file

Additional file 2 **Table S2. Inter-assay reproducibility of qPCR assay**. Click here for file
