# Supplementary material for: Comparison of diagnostic methods for the detection and quantification of the four sympatric Plasmodium species in field samples from Papua New Guinea
Source: Malar J. 2010 Dec 14;9:361. doi: 10.1186/1475-2875-9-361 (PMC3016373; doi:10.1186/1475-2875-9-361)
Supplement: Additional file 1 — Table S1. Effect of mixed-species infection on sensitivity of duplex qPCR. [file 1475-2875-9-361-S1.DOCX]

**Table 1. Effect of mixed-species infection on sensitivity of duplex qPCR**

**A. Duplex reaction *P. falciparum* & *P. vivax***

| Species diagnosed |  | **Ct**  no other template | **Ct**  both templates present | | |
| --- | --- | --- | --- | --- | --- |
|  | **Copy no.^1^** | **0** | **20** | **200** | **2000** |
| ***P. falciparum*** | **2000** | 29.8 | 29.9 | 29.6 | 29.3 |
|  | **200** | 33.1 | 33.5 | 32.9 | 32.8 |
|  | **20** | 37.3 | 36.7 | 36.1 | 38.1 |
| ***P. vivax*** | **2000** | 29.3 | 29.3 | 29.1 | 28.8 |
|  | **200** | 32.9 | 32.3 | 32.3 | 31.7 |
|  | **20** | 36.3 | 34.5 | 34.3 | 33.5 |

**B. Duplex reaction *P. ovale* & *P. malariae***

| Species diagnosed |  | **Ct**  no other species | **Ct**  both templates present | | |
| --- | --- | --- | --- | --- | --- |
|  | **Copy no.** |  | **20** | **200** | **2000** |
| *P. ovale* | **2000** | 30.5 | 30.3 | 30.4 | 30.3 |
|  | **200** | 34.0 | 33.8 | 33.7 | 33.5 |
|  | **20** | 37.5 | 36.5 | 36.9 | 39.4 |
| *P. malariae* | **2000** | 29.6 | 30.0 | 30.3 | 30.1 |
|  | **200** | 33.4 | 33.6 | 33.8 | 35.1 |
|  | **20** | 37.1 | 37.5 | 37.0 | >40^2^ |

^1^ Number of template copies (control plasmid) in reaction mix.

^2^ A Ct value of >40 was considered negative.
