# Supplementary material for: Comparison of diagnostic methods for the detection and quantification of the four sympatric Plasmodium species in field samples from Papua New Guinea
Source: Malar J. 2010 Dec 14;9:361. doi: 10.1186/1475-2875-9-361 (PMC3016373; doi:10.1186/1475-2875-9-361)
Supplement: Additional file 2 — Table S2. Inter-assay reproducibility of qPCR assay. [file 1475-2875-9-361-S2.DOCX]

**Table 2. Inter-assay reproducibility**

|  | **Log copies/ul** | **Mean Ct** | **SD^1^** | **CV (%)^2^** |
| --- | --- | --- | --- | --- |
| ***P. falciparum*** | **6** | 19,36 | 0,57 | 2,95 |
|  | **5** | 21,39 | 0,92 | 4,30 |
|  | **4** | 24,66 | 0,69 | 2,80 |
|  | **3** | 28,37 | 0,68 | 2,38 |
|  | **2** | 31,98 | 0,57 | 1,77 |
|  | **1** | 36,75 | 1,75 | 4,76 |
| ***P. vivax*** | **6** | 18,03 | 0,84 | 4,64 |
|  | **5** | 20,55 | 0,90 | 4,37 |
|  | **4** | 23,44 | 1,71 | 5 |
|  | **3** | 26,86 | 1,07 | 4,01 |
|  | **2** | 30,48 | 1,17 | 3,85 |
|  | **1** | 36,55 | 1,76 | 4,84 |
| ***P. malariae*** | **6** | 19,79 | 0,62 | 3,15 |
|  | **5** | 22,04 | 0,50 | 2,28 |
|  | **4** | 25,21 | 0,34 | 1,35 |
|  | **3** | 28,82 | 0,45 | 1,55 |
|  | **2** | 33,47 | 0,72 | 2,14 |
|  | **1** | 37,22 | 0,82 | 2,22 |
| ***P. ovale*** | **6** | 18,73 | 0,57 | 3,05 |
|  | **5** | 20,73 | 0,57 | 2,75 |
|  | **4** | 24,15 | 0,54 | 2,25 |
|  | **3** | 28,01 | 1,00 | 3,56 |
|  | **2** | 31,42 | 0,53 | 1,70 |
|  | **1** | 36,15 | 1,46 | 4,04 |

**^1^** Standard Deviation (SD)

**^2^** Inter-assay coefficient of variation **CV** = (SD/Mean)*100
